# Caffeine Compromises Proliferation of Human Hippocampal Progenitor Cells

**DOI:** 10.3389/fcell.2020.00806

**Published:** 2020-09-08

**Authors:** Vikki Houghton, Andrea Du Preez, Sophie Lefèvre-Arbogast, Chiara de Lucia, Dorrain Y. Low, Mireia Urpi-Sarda, Silvie R. Ruigrok, Barbara Altendorfer, Raúl González-Domínguez, Cristina Andres-Lacueva, Ludwig Aigner, Paul J. Lucassen, Aniko Korosi, Cécilia Samieri, Claudine Manach, Sandrine Thuret

**Affiliations:** ^1^Department of Basic and Clinical Neuroscience, Maurice Wohl Clinical Neuroscience Institute, Institute of Psychiatry, Psychology and Neuroscience, King’s College London, London, United Kingdom; ^2^University of Bordeaux, INSERM, BPH, U1219, Bordeaux, France; ^3^INRA, UMR 1019, Human Nutrition Unit, Université Clermont Auvergne, Clermont-Ferrand, France; ^4^Nutrition, Food Science and Gastronomy Department, Faculty of Pharmacy and Food Science, CIBER Fragilidad y Envejecimiento Saludable, Instituto de Salud Carlos III, University of Barcelona, Barcelona, Spain; ^5^Brain Plasticity Group, Swammerdam Institute for Life Sciences, Center for Neuroscience, University of Amsterdam, Amsterdam, Netherlands; ^6^Institute of Molecular Regenerative Medicine, Spinal Cord Injury and Tissue Regeneration Center Salzburg, Paracelsus Medical University, Salzburg, Austria; ^7^Department of Neurology, University Hospital Carl Gustav Carus, Technische Universität Dresden, Dresden, Germany

**Keywords:** adult hippocampal neurogenesis, diet, caffeine, hippocampal progenitor integrity, hippocampal progenitor proliferation

## Abstract

The age-associated reduction in the proliferation of neural stem cells (NSCs) has been associated with cognitive decline. Numerous factors have been shown to modulate this process, including dietary components. Frequent consumption of caffeine has been correlated with an increased risk of cognitive decline, but further evidence of a negative effect on hippocampal progenitor proliferation is limited to animal models. Here, we used a human hippocampal progenitor cell line to investigate the effects of caffeine on hippocampal progenitor integrity and proliferation specifically. The effects of five caffeine concentrations (0 mM = control, 0.1 mM ∼ 150 mg, 0.25 mM ∼ 400 mg, 0.5 mM ∼ 750 mg, and 1.0 mM ∼ 1500 mg) were measured following acute (1 day) and repeated (3 days) exposure. Immunocytochemistry was used to quantify hippocampal progenitor integrity (i.e., SOX2- and Nestin-positive cells), proliferation (i.e., Ki67-positive cells), cell count (i.e., DAPI-positive cells), and apoptosis (i.e., CC3-positive cells). We found that progenitor integrity was significantly reduced in supraphysiological caffeine conditions (i.e., 1.0 mM ∼ 1500 mg), but relative to the lowest caffeine condition (i.e., 0.1 mM ∼ 150 mg) only. Moreover, repeated exposure to supraphysiological caffeine concentrations (i.e., 1.0 mM ∼ 1500 mg) was found to affect proliferation, significantly reducing % Ki67-positive cells relative to control and lower caffeine dose conditions (i.e., 0.1 mM ∼ 150 mg and 0.25 mM ∼ 400 mg). Caffeine treatment did not influence apoptosis and there were no significant differences in any measure between lower doses of caffeine (i.e., 0.1 mM, 0.25 mM, 0.5 mM) – representative of daily human caffeine intake – and control conditions. Our study demonstrates that dietary components such as caffeine can influence NSC integrity and proliferation and may be indicative of a mechanism by which diet affects cognitive outcomes.

## Introduction

Adult hippocampal neurogenesis (AHN), the formation of new neurons from neural progenitor cells, has recently regained considerable attention, particularly in the human hippocampus (Kempermann et al., 2018; Lucassen et al., 2020). This highly vascularized “neurogenic niche” retains developmental signals and morphogens that influence cell proliferation, differentiation, and survival throughout life (Spalding et al., 2013; Gonçalves et al., 2016). The rates at which these processes occur have been associated with hippocampal-dependent learning and memory functions (Snyder et al., 2005; Clelland et al., 2009; Sahay et al., 2011) and this association is particularly interesting when considering aging and cognitive decline, during which hippocampal function typically deteriorates (Small et al., 2002). Moreover, neural progenitor proliferation declines in rodents as aging progresses (Heine et al., 2004; Rao et al., 2006) and this has been strongly correlated with impaired performance in spatial memory and learning tasks (Sahay et al., 2011; Villeda et al., 2011).

This association with cognitive decline presents AHN as a unique target for preventative interventions. Accordingly, rescuing later life neurogenesis has recently gained interest and a focus has been given to the factors that modulate neurogenesis (Baptista and Andrade, 2018). While neurogenesis is facilitated by the neurogenic niche, it is not only central nervous system-derived signals that influence AHN. Indeed, AHN is also modulated by both the external environment (Lledo et al., 2006) and the system milieu (Villeda et al., 2011; Yousef et al., 2019). For example, stress and sleep deprivation have been shown to reduce AHN (Gould et al., 1998; Hairston et al., 2005; Lucassen et al., 2010), while running increases neurogenesis (van Praag et al., 2005). Moreover, these environmental factors have been similarly correlated with spatial learning and memory (Nilsson et al., 1999; Oomen et al., 2010, 2014), highlighting the possibility of leveraging behavioral interventions to target the neurogenic process and, consequently, cognitive ability.

Diet is another environmental factor that has been shown to influence the neurogenic process (Stangl and Thuret, 2009; Miquel et al., 2018; Abbink et al., 2020). *Drosophila* research shows that nutritional factors can influence the exit of neural hippocampal progenitors from quiescence (Chell and Brand, 2010; Spéder and Brand, 2014), and other nutritional-based changes to the hippocampal progenitor pool have been likewise demonstrated across other species (Spéder et al., 2011; Sakayori et al., 2013; Cavallucci et al., 2016). For instance, in humans, the nutrient-sensing pathways: the mammalian target of rapamycin (mTOR), sirtuin, and insulin-like growth factor 1, have all been associated with hippocampal progenitor maintenance (de Lucia et al., 2020). However, the influence of nutrition and meal content on the hippocampal progenitor pool occurs in a complex manner, with the nature of change dependent on the food groups consumed. For instance, a high fat diet has been shown to decrease proliferation in rats (Lindqvist et al., 2006), while omega-3 fatty acids increase proliferation in lobsters (Beltz et al., 2007). Interestingly, these changes to proliferation directionally correspond with their associated cognitive outcomes, as omega-3 has been shown to improve cognitive outcomes, while high fat diets impair cognitive performance (Winocur and Greenwood, 2005; Fotuhi et al., 2009; Witte et al., 2009; Yam et al., 2019). Thus, the variable nature of meal content and its influence on proliferation may provide a flexible and unique mechanism of regulating the neurogenic process within the human population. However, further defining the dietary components that affect the neurogenic process and their direction of influence is crucial before such dietary-based interventions can be developed.

Caffeine, the most widely consumed psychostimulant in the world (Ferré, 2016), has been widely implicated as a cognitive modulator (Rosso et al., 2008; Glade, 2010). Caffeine consumption has traditionally been argued to produce health benefits on a neurological basis, including protection against cognitive decline in women aged over 65 years (Ritchie et al., 2007; Arab et al., 2011). However, we recently demonstrated a negative effect of caffeine on cognition, identifying caffeine as one of 22 metabolites predictive of cognitive decline in an aging population, over a 13-year period (Low et al., 2019). Further evidence to support a negative effect of caffeine comes from animal models that focus on hippocampal neuronal proliferation. Specifically, when administered chronically, physiologically relevant doses of caffeine decreased neuronal precursor proliferation in rats (Wentz and Magavi, 2009), which was further correlated with impaired hippocampal-dependent learning and memory (Han et al., 2007). However, due to *in vivo* imaging constraints (Ho et al., 2013), the effect of caffeine on human hippocampal progenitor proliferation has not yet been explored. With the mixed clinical evidence on the impact of caffeine on cognitive decline and its large-scale consumption worldwide, further investigation is warranted. Determining the effects of caffeine on proliferation and the neurogenic process overall, and ultimately cognition, will contribute to our understanding of how diet affects these phenomena, which could assist in the development of appropriate interventions.

Therefore, this study investigated the effects of caffeine on human hippocampal progenitor proliferation, focusing on hippocampal proliferation, progenitor integrity (i.e., maintenance of the stem cell pool and proliferative/differentiative capacity), and progenitor apoptosis. We used a human hippocampal progenitor cell line, for the first time, to investigate, (i) the effects of five caffeine concentrations, and (ii) the effects of acute and repeated exposure to caffeine – all on hippocampal progenitor integrity, proliferation and apoptosis.

## Materials and Methods

### Cell Line and Culture Conditions

The human fetal hippocampal multipotent progenitor cell line *HPC0A07/03* (HPC; ReNeuron Ltd., Surrey, United Kingdom) was used in all experiments as previously described (de Lucia et al., 2020; Smeeth et al., in press). Cells were acquired from 12-week old female fetal tissue in accordance with United States and United Kingdom ethical and legal guidelines. and transfected with the c-mycERTAM gene construct creating an immortalized cell line that proliferates in the presence of the synthetic drug 4-hydroxy-tamoxifen (4-OHT) and spontaneously differentiates in its absence. For further details see Supplementary Material.

HPCs were cultured in reduced modified medium (RMM), namely Dulbecco’s Modified Eagle’s Media/F12 (DMEM:F12, Sigma), supplemented with 0.03% human albumin solution (Zenalb), 100 μg/mL human apo-transferrin, 16.2 μg/mL human putrescine diHCl, 5 μg/mL human recombinant insulin, 60 ng/mL progesterone, 2 mM L-glutamine and 40 ng/mL sodium selenite. For proliferation, the medium also included 10 ng/mL human basic fibroblast growth factor (bFGF), 20 ng/mL human epidermal growth factor (EGF) and 100 nM 4-OHT. Cells were grown on tissue culture flasks (Nunclon, Denmark), incubated at 37°C, 5% CO2 and saturated humidity, and were routinely passaged at 80% confluency before being plated for experiments.

### Proliferation Assay

**FIGURE 1:**
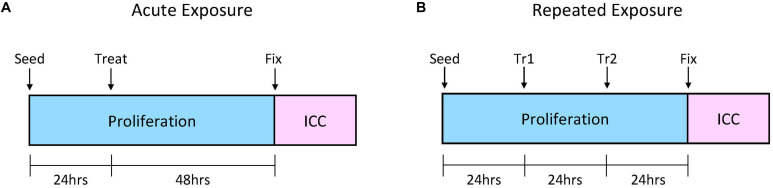
Schematic of the proliferation assays for the two caffeine exposure conditions. **(A)** Acute caffeine exposure. This plate received only one caffeine treatment, 24 h after seeding. Treatment involved a full replacement of culture medium with 100 μl caffeinated medium. **(B)** Repeated Caffeine Exposure. This plate received a treatment every 24 h after seeding. Treatment 1 (Tr1) involved a full replacement of culture medium with 100 μl caffeinated medium. Treatment 2 (Tr2) involved a “booster” treatment, where 20 μl of medium was removed and replaced with fresh caffeine medium. Booster treatments were made at 5× concentration. Both plates were fixed 72 h after seeding. ICC, immunocytochemistry. Cell line: *HPC0A07/03*. Passage number: P21; biological replicates: *n* = 3; technical replicates: *n* = 3.

The HPC proliferation assay was carried out as previously described (de Lucia et al., 2020; Smeeth et al., in press). Briefly, HPCs were seeded into two 96-well plates (Nunclon, Denmark) per experiment: one plate for acute (one-time) caffeine treatment, the other for repeated caffeine treatment. Plates were seeded at a density of 1.2 × 10^4^, at P21 in caffeine-free proliferation media, with three technical replicates and three biological replicates. All cells, excluding the control conditions, received caffeine treatment 24 h after seeding. Cells undergoing acute treatment were left undisturbed for 48 h, while cells undergoing repeated exposure received another caffeine treatment 24 h after the initial treatment. Control conditions were incubated in caffeine-free proliferation media in all instances. Seventy-two hours after seeding, all plates were washed and fixed as previously described (de Lucia et al., 2020; Smeeth et al., in press). Figure 1 depicts the assay timeline as per the two exposure conditions. For details on the proliferation assays and fixation methods see Supplementary Material.

### Caffeine Treatments

Caffeine (5 g) was obtained from Sigma (MO, United States) in powdered form, with a molecular weight of 194.19 g/mol. Caffeine conditions were as follows: control (no caffeine, media only); low (0.1 mM, ∼150 mg, ∼1 cup); moderate (0.25 mM, ∼400 mg, ∼2–3 cups); high (0.5 mM, ∼750 mg, ∼5 cups); and supraphysiological (1.0 mM, ∼1500 mg, ∼10 cups), reflecting human intake habits and previous animal models (Wentz and Magavi, 2009; Efsa Panel on Dietetic Products, Nutrition, and Allergies (NDA), 2015). Caffeine concentrations were calculated based on previous research stating that 150 mg of caffeine, the mean caffeine content of a Starbucks cappuccino (Ludwig et al., 2014), is approximately equivalent to 0.1 mM (Su et al., 2013a, b). For full details on the caffeine treatments see Supplementary Material.

### Immunocytochemistry

Cell count, progenitor cell integrity, progenitor proliferation, and cell death were visualized using 4′,6-diamidino-2-phenylindole (DAPI), Nestin and SRY-Box Transcription Factor 2 (SOX2), Ki67, and cleaved caspase-3 (CC3), respectively, using immunocytochemistry as previously described (de Lucia et al., 2020; Smeeth et al., in press). For protocol details, antibodies used, and representative images see Supplementary Figure S1.

### Image Analysis

Immunostainings were quantified using the semi-automated CellInsight NXT High Content Screening (HCS) platform (Thermo Fisher Scientific) and Studio Cell Analysis Software (Thermo Fisher Scientific), as previously described (de Lucia et al., 2020; Smeeth et al., in press). This platform relies on light intensity thresholds, which identify DAPI (wavelength 386) or secondary antibody fluorescence (wavelengths 488 and 555). These thresholds, combined with other parameters based on cell size and shape, identify cells stained by each antibody in an unbiased way and enable semi-automated quantification of immunocytochemical stains. Threshold settings were set by an author blinded to exposure/concentration and parameters were kept constant across experiments. For further details on the protocols and parameters used see Supplementary Material.

### Statistical Analyses

Data analyses were conducted using IBM SPSS Statistics 26 (IBM Ltd., Portsmouth, United Kingdom). All data were assessed for normality using probability-probability plots and the Kolmogorov–Smirnov test, and for homogeneity of variance using the Levene’s test. For data that did not conform to normality and/or homoscedasticity non-parametric statistical tests were applied. To evaluate differences between DAPI, Ki67, C33, and Ki67/CC3, a two-way analysis of variance (ANOVA) with a Bonferroni *post hoc* correction was applied. To evaluate differences in SOX2, Nestin, and Nestin/SOX2 a series of Kruskal–Wallis tests with Dunn’s *post hoc* corrections were applied. All tests carried out were two-sided and the alpha criterion used was *p* < 0.05. Data are represented as the mean (M) and standard deviation (SD), or the median (Mdn) and interquartile range (IQR).

## Results

### Exposure to Caffeine Reduces Cell Number

**FIGURE 2:**
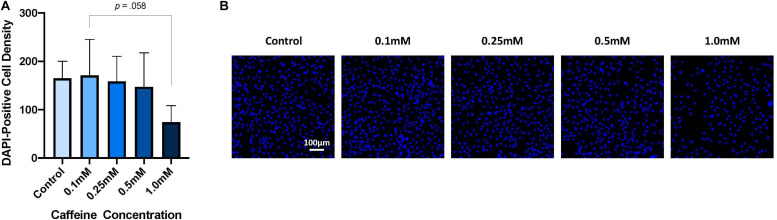
The effect of caffeine treatment on DAPI-positive cell density. **(A)** There was no significant interaction of caffeine concentration and exposure on DAPI-positive cell density [two-way ANOVA: *F*(4,20) = 0.179, *p* = 0.947], nor was there a significant main effect of exposure [*F*(1,20) = 0.317, *p* = 0.580]. However, caffeine had a significant main effect on DAPI-positive cell density [one-way ANOVA: *F*(4,25) = 3.041, *p* = 0.036]. *Post hoc* analyses revealed no significant differences in total cell number between any specific caffeine concentration and the control, nor between any caffeine concentrations. However, a statistically non-significant reduction in cell count was observed for the supraphysiological caffeine concentration (i.e., 1.0 mM, *M* = 74.13, SD = 34.27) compared with the lowest caffeine concentration (i.e., 0.1 mM, *M* = 171.12, SD = 74.21, *p* = 0.058). **(B)** Representative immunostaining, demonstrating DAPI-positive cell density following exposure to different caffeine concentrations. Images taken at 10× objective; scale bar represents 100 μm. Cell line: *HPC0A07/03*; passage number: P21; biological replicates: *n=3*; technical replicates: *n* = 3; data represents the mean (±SD); (adjusted *p-*values; Bonferroni correction). Graph not stratified by exposure given that no main effect of exposure was found. Graph presents the pooled data of acute and repeated exposure.

There was no significant interaction of caffeine concentration and exposure type, i.e., repeated versus acute caffeine treatment, on cell number, as measured by DAPI-positive cell density (*p* = 0.947), nor was there a significant main effect of exposure (*p* = 0.580). However, as shown in Figures 2A,B, there was a main effect of caffeine concentration on DAPI-positive cell density (*p* = 0.036), such that higher caffeine doses reduced cell number. However, due to issues of power, *post hoc* analyses revealed no specific differences between any of the caffeine conditions, but, although not statistically significant, an observed 58.6% reduction in cell count for the supraphysiological dose (i.e., 1.0 mM ∼ 1500 mg) relative to the lowest caffeine dose (i.e., 0.1 mM ∼ 150 mg) was seen (*p* = 0.058).

### Exposure to Supraphysiological Caffeine Concentrations Reduces Hippocampal Progenitor Integrity Compared With Lower Caffeine Doses Only

**FIGURE 3:**
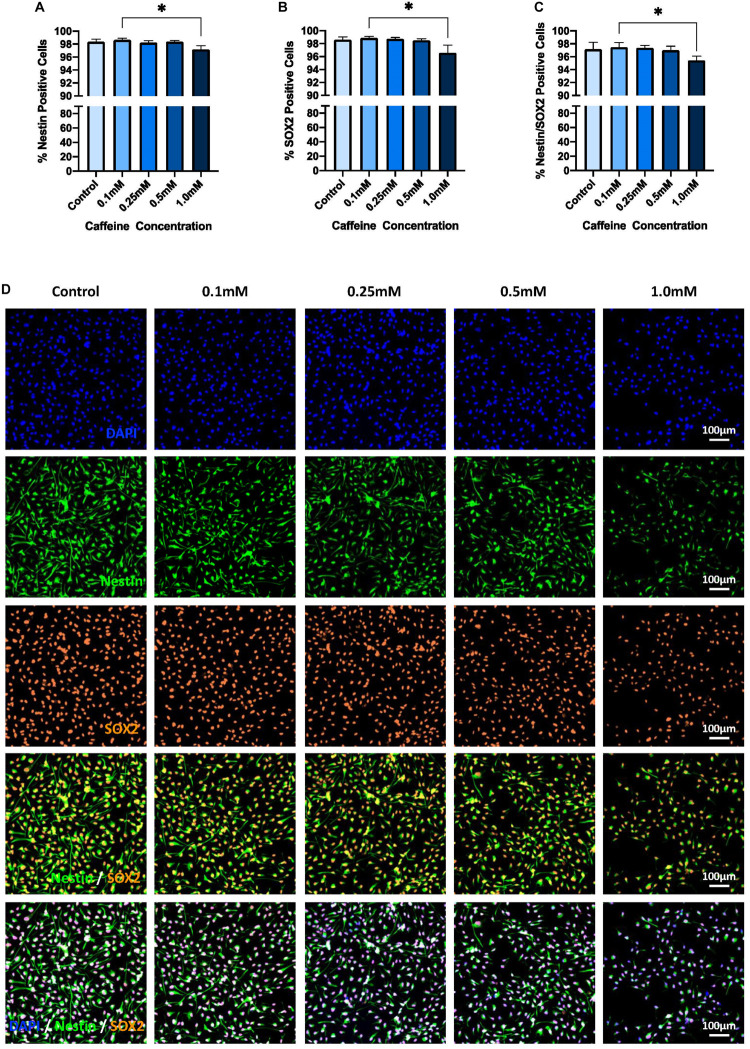
The effect of caffeine treatment on% Nestin-,% SOX2-, and Nestin/SOX2-positive cells. **(A)** There was no significant main effect of exposure on % Nestin-positive cells (Kruskal–Wallis test, *H* = 0.02, *df* = 1, *p* = 0.901) but there was a significant main effect of caffeine (Kruskal–Wallis test, *H* = 10.38, *df* = 4, *p* = 0.034). Dunn’s *post hoc* analyses revealed that the supraphysiological caffeine concentration (i.e., 1.0 mM; Mdn = 97.2, IQR = 1.99) had significantly reduced stem cell integrity compared with the lowest caffeine concentration (i.e., 0.1 mM, Mdn = 98.66, IQR = 0.72, *p* = 0.016). **(B)** There was no significant main effect of exposure on % SOX2-positive cells (Kruskal–Wallis test, *H* = 0.01, *df* = 1, *p* = 0.917) but there was a significant main effect of caffeine (Kruskal–Wallis test, *H* = 12.17, *df* = 4, *p* = 0.016). Dunn’s *post hoc* analyses revealed that the supraphysiological caffeine concentration (i.e., 1.0 mM; Mdn = 96.59, IQR = 2.8) was significantly reduced compared with the lowest caffeine concentration (i.e., 0.1 mM, Mdn = 98.9, IQR = 0.81, *p* = 0.013). **(C)** There was no significant main effect of exposure on % Nestin/SOX2-positive cells (Kruskal–Wallis test, *H* = 0.03, *df* = 1, *p* = 0.868) but there was a significant main effect of caffeine (Kruskal–Wallis test, *H* = 11.61, *df* = 4, *p* = 0.021). Dunn’s *post hoc* analyses revealed the supraphysiological caffeine concentration (i.e., 1.0 mM; Mdn = 95.46, IQR = 3.98) was significantly reduced compared with the lowest caffeine concentration (i.e., 0.1 mM, Mdn = 97.48, IQR = 1.11, *p* = 0.016). **(D)** Representative immunostaining, demonstrating, in order from the top panel, DAPI-positive cell density, % Nestin-, % SOX2-, and % Nestin/SOX2-positive cells following exposure to different caffeine concentrations. Images taken at 10× objective; scale bar represents 100 μm. % Nestin-, % SOX2-, and % Nestin/SOX2-positive cells are controlled for by DAPI. Cell line: *HPC0A07/03*; passage number: P21; biological replicates: *n* = 3; technical replicates: *n* = 3; Data represents the median (±IQR); ^∗^*p* < 0.05; (adjusted *p*-values; Dunn’s correction). Graphs not stratified by exposure given that no main effect of exposure was found. Graphs present the pooled data of acute and repeated exposure.

There was no significant main effect of exposure on hippocampal progenitor integrity, as measured by both % Nestin-positive (*p* = 0.901) and % SOX2-positive (*p* = 0.917) cells. However, as shown in Figure 3, there was a significant main effect of caffeine concentration on both % Nestin-positive (*p* = 0.034) and % SOX2-positive cells (*p* = 0.016), all while controlling for cell number.

*Post hoc* analyses revealed that the supraphysiological caffeine concentration (i.e., 1.0 mM ∼ 1500 mg) significantly reduced the % Nestin-positive cells by 1.5% relative to the lowest caffeine concentration (i.e., 0.1 mM ∼ 150 mg; *p* = 0.016; Figures 3A,D). No significant differences in % Nestin-positive cells for any of the caffeine concentrations relative to control were observed (0.1 mM ∼ 150 mg: *p* > 0.99; 0.25 mM ∼ 400 mg: *p* > 0.99; 0.5 mM ∼ 750 mg: *p* > 0.99; 1.0 mM ∼ 1500 mg: *p* = 0.388). However, it should be noted that the supraphysiological caffeine dose was reduced relative to control conditions but did not survive multiple comparison correction (non-adjusted *p* = 0.039).

Similar to Nestin data, *post hoc* analyses of % SOX2-positive cells revealed that the supraphysiological caffeinedose (i.e., 1.0 mM ∼ 1500 mg) significantly reduced % SOX2-positve cells by 2.3%, again, relative to the lowest caffeine concentration (0.1mM ∼ 150 mg, *p* = 0.013; Figures 3B,D). Moreover, there was an observed reduction, albeit not statistically significant, in % SOX2-positive cells in the supraphysiological caffeine dose relative to the moderate caffeine concentration, i.e., 0.25 mM ∼ 400 mg (*p* = 0.059). Again, no significant differences were observed relative to control conditions (0.1 mM ∼ 150 mg: *p* ≥ 0.99; 0.25 mM ∼ 400 mg: *p* > 0.99; 0.5 mM ∼ 750 mg: *p* > 0.99; 1.0 mM ∼ 1500 mg: *p* = 0.304). However, as with the Nestin data, % SOX2-positive cells in the supraphysiological caffeine condition were reduced relative to control but did not survive multiple comparison correction (non-adjusted *p* = 0.03).

Unsurprisingly, a similar results pattern was observed for % Nestin/SOX2-positve cells. Specifically, no significant main effect of exposure (*p* = 0.868) was observed, but there was a significant main effect of caffeine on % Nestin/SOX2-positive cells (*p* = 0.021), with the supraphysiological concentration reducing % Nestin/SOX2-positve cells by 2.1% relative to the lowest caffeine concentration only (*p* = 0.016; Figures 3C,D). Moreover, the % Nestin/SOX2-positive cells for the supraphysiological dose was reduced compared with the control condition (non-adjusted *p* = 0.029) and the moderate caffeine concentration, i.e., 0.25 mM ∼ 400 mg (non-adjusted *p* = 0.008) but these did not survive multiple comparison correction.

### Repeated Exposure to Supraphysiological Caffeine Concentrations Reduces Hippocampal Progenitor Proliferation

**FIGURE 4:**
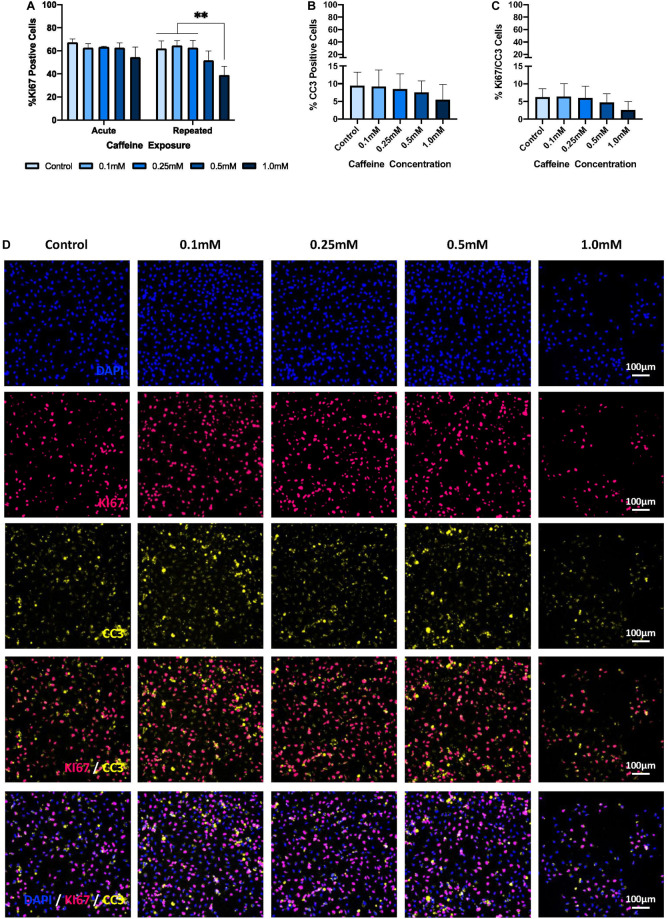
The effect of caffeine treatment on % Ki67-, % CC3-, and % Ki67/CC3-positive cells. **(A)** There was no significant interaction effect of caffeine concentration and exposure on % Ki67-positive cells [two-way ANOVA: *F*(4,20) = 2.235, *p* = 0.102]. However, a significant main effect of both exposure [two-way ANOVA: *F*(1,20) = 8.292, *p* = 0.009], and caffeine concentration [two-way ANOVA: *F*(4,20) = 9.81, *p* < 0.001] was found on % Ki67-positive cells. Specifically, Bonferroni *post hoc* analyses revealed that repeated treatment with the supraphysiological concentration (i.e., 1.0 mM; *M* = 39.05, SD = 7.5) significantly reduced proliferation compared with the control (*M* = 61.94, SD = 6.69, *p* = 0.001), the lowest caffeine dose (i.e., 0.1 mM; *M* = 64.580, SD = 4.403, *p* < 0.001), and the moderate caffeine dose (i.e., 0.25 mM; *M* = 62.68, SD = 6.35, *p* = 0.001). **(B)** There was no significant main effect of caffeine [one-way ANOVA: *F*(4,25) = 9.09, *p* = 0.474], exposure [two-way ANOVA: *F*(1,20) = 0.331, *p* = 0.571], nor an interaction effect [two-way ANOVA: *F*(4,20) = 0.677, *p* = 0.616] on apoptosis, that is % CC3-positive cells. **(C)** There was no significant main effect of caffeine [one-way ANOVA: *F*(4,25) = 1.767, *p* = 0.167], exposure [two-way ANOVA: *F*(1,20) = 0.097, *p* = 0.759], nor an interaction effect [two-way ANOVA: *F*(4,20) = 0.413, *p* = 0.797] on % CC3/Ki67-positive cells. **(D)** Representative immunostaining, demonstrating, in order from the top panel, DAPI-positive cell density, % Ki67-, % CC3-, and % Ki67/CC3-positive cells following exposure to different caffeine concentrations. Images taken at 10× objective; scale bar represents 100 μm. % Ki67-, % CC3-, and % Ki67/CC3-positive cells are controlled for by DAPI. Cell line: *HPC0A07/03*; passage number: P21; biological replicates: *n* = 3; technical replicates: *n* = 3; data represents the mean (±SD); ^∗∗^*p* < 0.01 (adjusted *p*-values; Bonferroni correction). Graphs **(B,C)** are not stratified by exposure given that no main effect of exposure was found. Graphs **(B,C)** present the pooled data of acute and repeated exposure.

There was no significant interaction effect of caffeine and exposure on proliferation, as measured by the percentage of Ki67-positive cells (*p* = 0.102). However, as shown in Figures 4A,D, there was both a significant main effect of exposure (*p* = 0.009) and caffeine concentration (*p* < 0.001) on the % Ki67-positive cells, all while controlling for cell number. Specifically, repeated exposure to the supraphysiological caffeine concentration (i.e., 1.0 mM ∼ 1500 mg) significantly reduced proliferation by 37% relative to control conditions (*p* = 0.001), by 39.5% relative to the lowest caffeine dose (i.e., 0.1 mM ∼ 150 mg; *p* < 0.001), and by 37.7% relative to the moderate caffeine dose (i.e., 0.25 mM ∼ 400 mg; *p* = 0.001). No significant differences were found between the control condition and the other caffeine concentrations, i.e., 0.1 mM (*p* > 0.99), 0.25 mM (*p* > 0.99), and 0.5 mM (*p* = 0.446), nor were any significant differences observed for acute exposure, that is, a single, one-time caffeine treatment.

### Exposure to Caffeine Does Not Affect Apoptosis

As depicted in Figures 4B,D, there was no significant interaction of caffeine concentration and exposure on apoptosis, as measured by % CC3-positive cells (*p* = 0.616), nor was there a significant main effect of exposure (*p* = 0.571) or caffeine concentration (*p* = 0.474) – all while controlling for cell number. Furthermore, as shown in Figures 4C,D, there was no significant interaction of caffeine concentration and exposure on proliferative cell death, as measured by % Ki67/CC3-positive cells (*p* = 0.797), nor was there a significant main effect of exposure (*p* = 0.759) or caffeine concentration (*p* = 0.167) – again, all while controlling for cell number.

## Discussion

In this study we explore the effects of acute and repeated caffeine exposure at different concentrations on hippocampal progenitor proliferation, integrity, and apoptosis, using an *in vitro* hippocampal cellular model. We demonstrate that a repeated supraphysiological dose of caffeine, i.e., 1.0 mM ∼ 1500 mg or ∼10 cups of coffee, significantly reduces progenitor proliferation, as measured by % Ki67-positive cells, relative to the control condition (no caffeine) and to both the lowest (i.e., 0.1 mM ∼ 150 mg or ∼1 cup) and moderate (i.e., 0.25 mM ∼ 400 mg or 2–3 cups) caffeine concentrations. Moreover, the supraphysiological dose (∼10 cups of coffee), whether acutely or repeatedly administered, negatively influences progenitor integrity, as measured by both % Nestin- and % SOX2-positve cells, but only when compared with the lowest caffeine dose (∼1 cup of coffee). Finally, we show that caffeine, irrespective of the degree of exposure or concentration, does not affect overall, or proliferative, cell death, as measured by % CC3-positive cells and % Ki67/CC3-positive cells, respectively.

Our finding that repeated treatment with a supraphysiological caffeine concentration, that is, intake of ∼10 cups of coffee, reduces hippocampal progenitor proliferation directly contrasts previous findings from Wentz and Magavi (2009), who used an animal model and observe that supraphysiological doses increase proliferation. However, this inconsistency could be attributed to differences in study design; all previous findings were from an animal model, and therefore are not entirely translatable to our own study design that uses a human *in vitro* cellular model. Furthermore, our study found no effect of lower caffeine doses on hippocampal proliferation, despite previous literature demonstrating a decrease in proliferation (Han et al., 2007; Wentz and Magavi, 2009). While the discrepancies between our findings and that of the previous literature could be a consequence of the different models used, it is more likely attributable to the different timescales investigated. While our study investigated repeated exposure over 72-h of proliferation, Wentz and Magavi (2009) and Han et al. (2007) investigated caffeine exposure over 7 days and 4 weeks, respectively. In the context of our work, while the supraphysiological caffeine concentration is strong enough to produce a detrimental effect over a short period of time, our 72-h paradigm may be insufficient to replicate the results seen from chronic exposure with lower, more physiologically relevant doses. Therefore, future work should seek to extend our paradigm to explore the longer-term effects of chronic, rather than repeated treatment, with physiologically relevant caffeine concentrations.

Previously unexplored within an *in vitro* model of HPCs, our findings relating to % Nestin- and % SOX2-positive cells may provide some insight into the mechanisms by which the supraphysiological caffeine dose influences proliferation. Used as markers for progenitor integrity in this study, Nestin and SOX2 represent the maintenance of the stem cell pool, with their knockout having been shown to lead to a reduction in total neural stem cell (NSC) quantity (Favaro et al., 2009; Park et al., 2010). In particular, SOX2 has been implicated as an important requirement for the maintenance of self-renewal and pluripotency in human embryonic hippocampal progenitors (Fong et al., 2008), and this has been further demonstrated in adult neural hippocampal progenitors. Ferri et al. (2004) found that knocking down SOX2 leads to reduced proliferation and a depletion of the neural hippocampal progenitor pool – a finding seemingly consistent with our own. Indeed, we report a reduction in both % Nestin-, % SOX- and % Nestin/SOX2-positive cells, and simultaneously find no change in either the total % CC3-positive cells or % CC3/Ki67-positive cells (i.e., specifically proliferative cell death), suggesting that the observed decrease in proliferation following repeated supraphysiological caffeine treatment could stem from a reduction in the hippocampal progenitor pool itself. However, given that Ki67 was not co-labeled with SOX2, this requires further substantiation. We do not know if the observed reduction in % Ki67-positive cells derive directly from the observed decrease in % SOX2-positive cells. It is possible that the two may be independent; our findings for proliferation could pertain to a reduction in the proliferative capacity and/or speed of NSCs rather than as a knock-on effect of a reduced progenitor pool.

Interestingly, we find no statistically significant effect of supraphysiological caffeine doses on SOX2 relative to control conditions, however we believe that this could potentially be due to issues of power (Cremers et al., 2017), given that prior to *post hoc* adjustment, the supraphysiological concentration of ∼10 cups of coffee shows a reduction in both % Nestin-, % SOX2-, and % Nestin/Ki67-positve cells, all relative to control conditions. Furthermore, it is notable that hippocampal progenitor integrity was statistically assessed using non-parametric methods, which are typically less powerful than parametric equivalents (Siegel, 1957). Therefore, it would be highly profitable for future research to include a greater sample size to more fully elucidate the effect of supraphysiological caffeine concentrations on hippocampal progenitor integrity.

The precise mechanisms by which caffeine affects proliferation are widely unknown, but the observed changes to % SOX2-positive cells may provide some insight. Caffeine has commonly been associated with protein kinase B (PKB or Akt) signaling; specifically, it has been attributed to downregulating Akt signaling in a wide range of cell types, from HeLa to mouse epidermal cell lines (Nomura et al., 2005; Saiki et al., 2011). Pertinently, Akt signaling has been linked with SOX2, having been shown to promote the expression of SOX2 adult hippocampal neural progenitor cells (Peltier et al., 2010). Furthermore, Akt signaling itself decreases with age, akin to SOX2 expression and neurogenesis overall, but its reactivation has been shown to ameliorate age-related defects in neuronal development (Tang et al., 2019). It is therefore possible that our finding of reduced%SOX2-positve cells following supraphysiological caffeine treatment is a product of downregulated Akt signaling. To our knowledge, the effect of caffeine on Akt signaling within an HPC cell line has not yet been investigated, and therefore future research would be instrumental in validating a link between caffeine and SOX2 expression in HPCs and revealing whether this action could be mediated by Akt signaling.

While our work reveals a negative effect of supraphysiological caffeine on human hippocampal progenitor integrity and proliferation, there are some limitations in that our model may have influenced the extent to which caffeine affects this process. For example, caffeine is metabolized in the liver by the enzyme CYP1A2, which accounts for approximately 90% of caffeine metabolism (Arnaud, 2011). Interestingly, a C/A polymorphism in intron 1 of the CYP1A2 gene appears to affect CYP1A2 enzymatic activity, and ultimately alter the rate of caffeine metabolism (Sachse et al., 2001). Indeed, Butler et al. (1992) defined CYP1A2 activity as being trimodally distributed, with slow, intermediate, and rapid metabolizers, as determined by caffeine urinary metabolite analyses. Essentially, the rates of caffeine clearance differ depend on an individual’s genetic variant, and therefore the amount of time that caffeine is present in the systemic environment is subject to interindividual differences. These differences in caffeine metabolic rates have been associated with differences in the risk of some neurodegenerative diseases, with individuals possessing the C allele, i.e., slow metabolizers, having decreased caffeine-related risk of Parkinson’s Disease (Popat et al., 2011; Chuang et al., 2016). Therefore, it is possible that this polymorphism may also mediate differences in the way caffeine affects proliferation, especially considering that caffeine reaches the brain via the systemic environment. Our study measures the direct effect of caffeine exposure on hippocampal progenitor cells, without accounting for differential metabolic rates in the liver caused by the CYP1A2 polymorphism.

Furthermore, while the caffeine concentrations used in our study reflect “intake,” this is not representative of peak plasma levels obtained following caffeine metabolism. Indeed, around 99% of caffeine is metabolized into paraxanthine, theobromine, and theophylline (Arnaud, 1993; Nehlig, 2018) and, thus, only residual caffeine remains in the systemic environment. For instance, consumption of 160 mg of caffeine, in the form of a hot coffee, was shown to produce an average peak plasma level of 3.74 μg/mL, or 19.26 μM, in humans (White et al., 2016). The lowest caffeine concentration in our study, 0.1 mM, represents approximately 150 mg of caffeine (Su et al., 2013a, b), or one Starbucks cappuccino (Ludwig et al., 2014), whereas plasma caffeine levels typically reach between 20 and 50 μM (Graham, 2001). Therefore, the levels of caffeine tested in this study reflect supranutritional doses, not the physiologically relevant concentrations that would reach the neurogenic niche *in vivo*. However, this study provides proof of concept that caffeine can modulate hippocampal progenitor proliferation, but it would be profitable for future research to investigate the effects of nutritional and supranutritional caffeine concentrations on this process over time. Understanding when in the trajectory of the neurogenic process these changes occur would be hugely beneficial for developing more targeted prevention strategies. Applying a growth curve analysis strategy to the proliferation and differentiation assays could be a viable solution.

An additional limitation to our work is that although CC3 is a commonly used maker for apoptosis, there are multiple modes of cell death, and even several pathways of apoptotic cell death (Galluzzi et al., 2018) that cannot be captured by a single marker, and particularly under stressful conditions *in vivo* (Riegelsberger et al., 2011). Thus, although we observe no change in apoptosis in the context of our work, additional makers (e.g., Annexin and TUNEL) would provide a more comprehensive overview of the apoptotic process.

Furthermore, although our aim was to explore the effect of caffeine on hippocampal progenitor cells, given that diet has been shown to specifically influence neural hippocampal progenitor behavior (Spéder et al., 2011; Sakayori et al., 2013), our work would hugely benefit from exploring the impact of caffeine on neural progenitor differentiation. By only investigating proliferation, we do not know what longer-term, knock-on effects might arise from caffeine treatment, with respect to differentiation and/or survival. Given that early changes to the hippocampal progenitor pool can reduce neurogenesis and result in morphological abnormalities of the resulting neurons (Cavallaro et al., 2008), future work should seek to extend our paradigm to also evaluate the impact of caffeine on differentiation and neuron morphology in order to more fully capture the impact of caffeine on the neurogenic process as a whole.

Finally, it is important to acknowledge that cell models are somewhat removed from an *in vivo* system making it challenging to account for any organism-wide changes. Furthermore, our model specifically is hindered by the lack of microglia, which play a key role in NSC regulation (Cunningham et al., 2013; Liu et al., 2013) and by the use of fetal NSCs to study proliferation during later life stages. Additionally, our findings should be interpreted with caution when generalizing to both male and females, given that this is a female cell line. Research shows sexual dimorphism in cognition (Ycaza Herrera et al., 2019), caffeine metabolism (Adan et al., 2008; Denden et al., 2016) and neurogenesis (Greiner et al., 2019).

However, despite these limitations to our work, this study is the first, to our knowledge, to investigate the direct effects of caffeine on hippocampal progenitor proliferation and integrity using a human *in vitro* cellular model. NSC proliferation is influenced by a range of systemic and environmental factors that are difficult to control for in an *in vivo* environment (Azari and Reynolds, 2016) – an issue that is mostly controlled for in *in vitro* models. Moreover, species and strain differences have long been a criticism of animal models (Martić-Kehl et al., 2012), and proliferation in particular has been shown to widely differ amongst mammalian species (Amrein et al., 2011). Therefore, our work investigates the direct effect of caffeine on human hippocampal stem cell proliferation and is thus likely to yield results with greater translational value.

Additionally, it is worth mentioning that our results have implications beyond the impact of metabolites or drugs on learning, aging, and cognitive decline. Indeed, recent research has highlighted the interaction of certain drugs with NSCs (Ikhsan et al., 2019), something with which our results are in strong accordance. Notably, there is a strong evidence base demonstrating a clear interaction of antidepressants with NSCs (Santarelli et al., 2003; Anacker et al., 2011), producing an increase in hippocampal progenitor proliferation. However, there is limited data available for substances beyond antidepressants (Ikhsan et al., 2019), especially concerning those of a non-pharmacological nature. Therefore, not only do our findings contribute to the growing discussion surrounding drug-NSC interactions, but they also provide evidence of such an interaction with a dietary-based substance, highlighting the possibility of utilizing diet as a non-pharmacological intervention to positively influence hippocampal neurogenesis. However, further research is required to identify positive dietary components and fully elucidate their interaction with NSCs and their effect on hippocampal neurogenesis.

## Conclusion

In summary, our study demonstrates that dietary components such as caffeine can influence hippocampal progenitor proliferation and may be indicative of one mechanism by which diet affects cognitive outcomes. However, future research that (i) further explores the effects of human consumption-related caffeine doses on both neural progenitor proliferation and differentiation, and (ii) correlates this with cognitive outcomes, are now needed.

## Data Availability Statement

All datasets presented in this study are included in the article/Supplementary Material.

## Author Contributions

VH: design and conceptualization of the study, data collection, statistical analysis, data interpretation, and drafting and revising the manuscript for intellectual content. AD: design and conceptualization of the study, data collection, supervision, statistical analysis, data interpretation, and drafting and revising the manuscript for intellectual content. SL-A: statistical analysis, data interpretation, and revising the manuscript for intellectual content. CL, DL, MU-S, SR, BA, RG-D, CA-L, LA, PL, AK, CS, and CM: data interpretation and revising the manuscript for intellectual content. ST: design and conceptualization of the study, statistical analysis, data interpretation, and drafting and revising the manuscript for intellectual content. All authors contributed to the article and approved the submitted version.

## Conflict of Interest

The authors declare that the research was conducted in the absence of any commercial or financial relationships that could be construed as a potential conflict of interest.
