# Supplementary material for: Caffeine Compromises Proliferation of Human Hippocampal Progenitor Cells
Source: Front Cell Dev Biol. 2020 Sep 8;8:806. doi: 10.3389/fcell.2020.00806 (PMC7505931; doi:10.3389/fcell.2020.00806)
Supplement: Supplementary file 1 [file Data_Sheet_1.pdf]

Supplementary Material

1 Supplementary Methods

1.1 Cell line

The human fetal hippocampal multipotent progenitor cell line *HPC0A07/03* (HPC; ReNeuron Ltd, Surrey, UK) was used in all experiments as previously described (de Lucia et al., 2020; Smeeth et al., 2020). Cells were infected with a retroviral vector pLNCX2, encoding the c-MycERTAM transgene construct to generate a conditionally immortalised cell line. This transgene is solely activated by the synthetic steroid 4-hydroxytamoxifen (4-OHT) and as such cells can be continuously cultured without differentiating in the presence of 4-OHT, basic fibroblast growth factor (bFGF) and epidermal growth factor (EGF). Removal of 4-OHT, bFGF and EGF from the culture medium allows the cells to differentiate into neurons, oligodendrocytes and astrocytes. Based on previous work within the lab, seven days of differentiation results in a cell population composed of 35% doublecortin (DCX)-positive immature neuroblasts, 25% microtubule associated protein 2 (MAP2)-positive neurons, 27% S100b-positive astrocytes and 2% O1-positive oligodendrocytes (Anacker et al., 2011). The remaining population maintain a neural progenitor cell phenotype.

1.2 Proliferation Assay

The hippocampal progenitor cell (HPC) proliferation assay was carried out as previously described (de Lucia et al., 2020; Smeeth et al., 2020). Briefly, HPCs were seeded into two 96-well plates (Nunclon, Denmark) per experiment: one plate for acute (one-time) caffeine treatment, the other for repeated caffeine treatment. Plates were seeded at a density of 1.2 x 10^4, at P21, with three technical replicates and three biological replicates. After seeding, cells were cultured in proliferation medium (reduced modified medium (RMM), with human EGF, human bFGF, and 4-OHT for 24 hours, after which the media was replaced by caffeine supplemented proliferation media (i.e., full treatment) for 48 hours. The acute treatment condition was left undisturbed for 48 hours, while the repeated condition received another caffeine treatment 24 hours later, where 20µl of the caffeine supplemented proliferation media was removed and replaced with a concentrated caffeine dose (i.e., booster treatment). Control conditions were incubated in caffeine-free proliferation media in all instances. Seventy-two hours after seeding, plates were washed with phosphate buffered saline (PBS) and fixed with 4% paraformaldehyde (PFA) for 20 minutes at room temperature. Post-fixation, plates were washed and stored in PBS with sodium azide (NaN3; 0.05% dilution) at 4°C in preparation for immunocytochemistry.

1.3 Caffeine Treatments

Prior to experimentation, caffeine concentrations were measured and dissolved in proliferation medium (RMM, with EGF, bFGF, and 4-OHT) separately for the two treatment types (i.e.., full and booster). As full treatments involved completely replacing the proliferation media, these were made at 1x the intended concentration. For repeated exposure, booster treatments at 5x the intended concentration were used, and 20µl of the total 100µl media was replaced to prevent interfering with the cell-produced factors. All caffeine concentrations (i.e., full and booster) and control proliferation media were clarified, aliquoted and frozen at -4°C for future use. Since caffeine content has been shown to differ depending on brewing method (Bell et al., 1996), to minimize any changes in caffeine concentration, all caffeine and control aliquots were defrosted in the 37°C water bath for ten minutes and clarified before use. All aliquots were thawed only once prior to use.

1.4 Immunocytochemistry

To quantify proliferation and progenitor cell integrity, immunocytochemistry was used as previously described (de Lucia et al., 2020; Smeeth et al. 2020). Briefly, PFA-fixed cells were incubated for 1h at room temperature in blocking solution, consisting of PBS-NaN3 with 5% normal donkey serum (D9963, Sigma) and 0.3% Triton-X (93443, Sigma). Cells were then incubated in 30µl of primary antibodies: Ki67 for proliferation, (Mouse anti-Ki67, 1:800, Cell Signalling Technology), Cleaved Caspase 3 for apoptosis (CC3; rabbit anti-CC3, 1:500, Cell Signalling Technology), and Nestin and SOX2 for hippocampal progenitor integrity (Mouse anti-Nestin, 1:1000, Merck Millipore; Mouse anti-Nestin, 1:1000, Abcam) - all diluted in blocking solution overnight at 4°C. Cells were then washed twice with PBS, and incubated in blocking solution for 30 minutes at room temperature, before being incubated in 30µl of secondary antibodies (Alexa 488 donkey anti-mouse, 1:500, Life Technologies, A-21202; Alexa 555 donkey anti-rabbit, 1:500, Life Technologies, A-31572), diluted in blocking solution, for 2h at room temperature in the dark. Cells were then washed twice with PBS and incubated at room temperature for 5 minutes in 50µl of 300µm 4′,6-diamidino-2-phenylindole solution diluted in PBS (DAPI; D9542-5mg, Sigma). Finally, cells were washed twice more before being stored in 200µl PBS with NaN3 (0.05% dilution) at 4°C in the dark.

1.5 Image Analysis

In brief, images were taken with a 10x objective that autofocuses using the DAPI stain. Exposure time was manually defined to ensure a good signal to noise ratio. Individual cells were determined using the DAPI-positive nuclei, and smoothing, threshold, and segmentation parameters were modified as required to ensure accurate outlining of nuclei. Consequently, DAPI stains too small or large to be counted as nuclei were excluded. Cellular markers of interest were then identified and quantified, with defined parameters that were dependent on each marker's location in relation to the nucleus. Nuclear proteins were indicated using a round target that overlaid the nuclear outline. Conversely, cytoplasmic proteins were indicated using a donut target, where the nucleus was denoted with the target's inner boundary. Average intensity thresholds (AIT) were manually defined, to distinguish specific fluorescent signals from unspecific binding and background noise. To set these, the experimental wells were compared to the negative control wells (i.e., without primary antibodies), and the threshold was set above the highest intensity signal of the negative control. Consequently, the positive cells (those with an AIT above the set threshold) could be differentiated from the negative cells (AIT below the set threshold). These parameters were kept constant throughout every experiment. Once the parameters had been set, plates were scanned. Fifteen fields per well were captured, in which the software calculates the percentage of all cells positive for each marker (identified by the appropriate cellular stain), as controlled for by DAPI-positive nuclei. This quantified 4000–10,000 cells per well depending on the treatment used.

2

2 Supplementary Figures

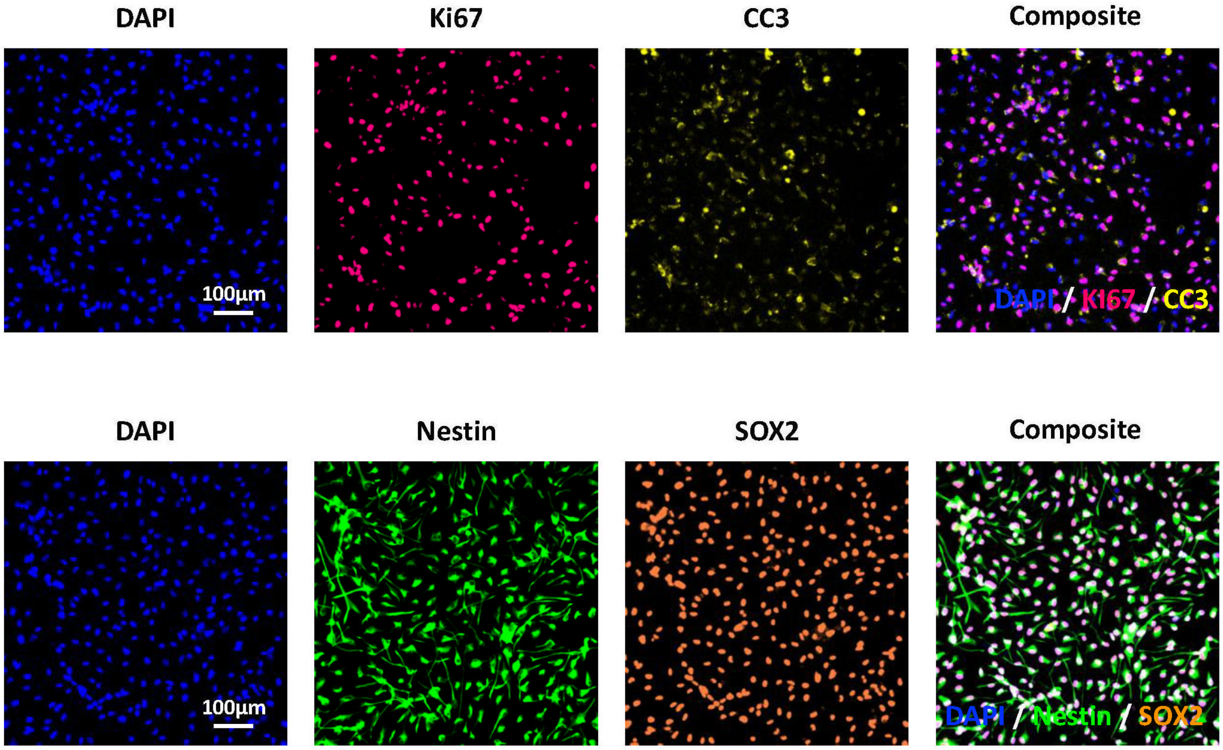

<u>Figure S.1</u>. Representative images of immunostaining. Each box represents a field analysed by the CellInsight software. Row 1 represents (from left to right) DAPI in blue, Ki67 in pink, CC3 in yellow, and the composite image. Row 2 represents (from left to right) DAPI in blue, Nestin in green, SOX2 in orange, and the composite image. Images taken with at 10x objective; scale bar, on the bottom left box, represents 100µm. Cell line: *HPC0A07/03*; Passage number: P21.

3 Supplementary References

Anacker, C., Zunszain, P. A., Cattaneo, A., Carvalho, L. A., Garabedian, M. J., Thuret, S., et al. (2011). Antidepressants increase human hippocampal neurogenesis by activating the glucocorticoid receptor. *Mol. Psychiatry* 16, 738–750. doi:10.1038/mp.2011.26.

Bell, L. N., Wetzel, C. R., and Grand, A. N. (1996). Caffeine content in coffee as influenced by grinding and brewing techniques. *Food Res. Int.* 29, 785–789. doi:https://doi.org/10.1016/S0963-9969(97)00002-1.

de Lucia, C., Murphy, T., Steves, C. J., Dobson, R. J. B., Proitsi, P., and Thuret, S. (2020). Lifestyle mediates the role of nutrient-sensing pathways in cognitive aging: cellular and epidemiological evidence. *Commun. Biol.* 3. doi:10.1038/s42003-020-0844-1.

Smeeth, D. M., Kourouzidou, I., Duarte, R. R. R., Powell, T. R., and Thuret, S. (2020). Prolactin, Estradiol and Testosterone Differentially Impact Human Hippocampal Neurogenesis in an In Vitro Model. *Neuroscience*, 1–25. doi:10.1016/j.neuroscience.2019.12.021.
